# Ultrafiltration of Whole Milk: Impact of Homogenization and Ultrafiltration Temperature on Processing Efficiency and Component Retention

**DOI:** 10.3390/foods14081370

**Published:** 2025-04-16

**Authors:** Pramith U. Don, Zeel Modi, Kartik Shah, Prafulla Salunke

**Affiliations:** 1 Dairy and Food Science Department, Midwest Dairy Foods Research Centre, South Dakota State University, Brookings, SD 57007, USA; pramithmanosha.ukwattagedon@jacks.sdstate.edu (P.U.D.); zeel.modi@sdstate.edu (Z.M.); 2 Idaho Milk Products, Jerome, ID 83338, USA; 3 Sargento Foods, Plymouth, WI 53073, USA

**Keywords:** whole milk, ultrafiltration, homogenization, temperature and minerals

## Abstract

The objective of this study was to investigate the impact of thermal and homogenization pre-treatments on ultrafiltration (UF) efficiency and component retention in whole milk (WM). Four milk treatments were examined using a benchtop Optisep filtration system: skim milk (SM) processed in UF at 15 °C, homogenized WM processed in UF at 15 °C (T1), non-homogenized WM processed in UF at 15 °C (T2), and non-homogenized WM processed at 43 °C (T3). UF was performed using 10 kDa membranes to achieve 3× concentration, and the retention and flux rates were compared across treatments. Compositional changes were analyzed at each stage: initial, retentate (2X, 3X, Final), and permeate. The permeate flux varied across treatments; SM showed the highest initial permeate flux and achieved the target concentration in a shorter time. T1 maintained a steady permeate flux over time. T2 exhibited a steep decline in flux, reaching only a 2.5× concentration. In contrast, T3 initially displayed a higher permeate flux due to heat treatment and reduced viscosity. There were significant differences in compositional parameters. T1 retentate had significantly (*p* < 0.05) higher crude protein (CP), Ca, Mg, and Zn retention. Higher total solids (TS), non-protein nitrogen (NPN), and non-casein nitrogen (NCN), K, and P content were found in T3 permeate. The results from this study demonstrate that homogenization and heat treatment significantly impact the UF performance of WM, offering valuable approaches for achieving dairy product composition.

## 1. Introduction

Membrane separation is a physical method of separating milk components based on their size and molecular weight. Membrane processing offers distinct advantages in the dairy industry, including reduced energy consumption, greater processing capacity, and improved product [1]. Ultrafiltration (UF) is a widely used membrane filtration method in the dairy and beverage sector, enabling the selective retention and passage of certain components through a semi-permeable membrane. This membrane typically has pore sizes ranging from 0.01 to 0.1 µm, which selectively allow smaller particles, such as water, lactose, soluble minerals, non-protein nitrogen (NPN), and water-soluble vitamins, to pass through as permeate while retaining larger molecules, resulting in concentrated retentate [2]. As a result, the UF of milk yields a retentate enriched in proteins, fat, and colloidal minerals, with concentrations corresponding to the volume of permeate removed [3]. The UF concentrates protein in the same ratio as found in the milk from which it was processed. Therefore, the UF process facilitates the separation of components based on molecular size, making it ideal for applications requiring higher protein content and reduced lactose, such as cheese, high-protein yogurts, and milk protein concentrate (MPC).

Milk contains essential macro-minerals such as calcium (Ca), magnesium (Mg), potassium (K), sodium (Na), and phosphorus (P), along with trace minerals like sulfur (S), zinc (Zn), copper (Cu), Iron (Fe), and Manganese (Mn), which play a crucial role in nutritional and functional properties. The UF process impacts the distribution and retention of these minerals, with colloidal minerals like Ca and P primarily retained in the retentate due to their association with casein micelles, whereas soluble minerals such as Na and K tend to permeate through UF membranes; however, it also depends on membranes with a molecular weight cut-off [4]. The retention of Ca and P is particularly significant in dairy applications as they contribute to structural and textural properties in cheese, yogurt, and other concentrated dairy products [5]. The balance of minerals in retentate or permeate affects the functional properties of the products they are used in. A comprehensive understanding of mineral retention during UF is essential for optimizing dairy formulations to enhance nutritional value and product functionality.

The effectiveness of UF depends on parameters such as permeate flux, fouling resistance, and solute rejection [6]. These parameters are significantly influenced by critical factors, including membrane selection, which determines the separation efficiency; hydrodynamic conditions, such as flow velocity and turbulence; and feed composition [7]. The practical application of UF at the industrial level faces challenges such as concentration polarization and membrane fouling, where solutes accumulate on the membrane surface and within its pores, thereby reducing efficiency. Understanding the efficiency of protein separation during UF becomes crucial, as it influences the quality of dairy products as protein retention increases yields, benefiting manufacturers financially while also enhancing the functional properties and texture profile of dairy products [8,9].

UF has been widely studied for its ability to modify milk composition and enhance the functional properties of dairy products. UF concentrates milk proteins and fat. However, this concentration process also shifts the distribution of casein micelles’ size towards smaller micelles in the concentrated milk [10]. Furthermore, UF influences the acid gelation characteristics of milk due to whey protein denaturation and casein micelle interactions [11]. These differences are attributed to UF-induced modifications in the denaturation of whey protein and the association of casein micelles. The use of UF in cheese production offers various benefits. The use of UF in cheese production previously affected the white-brined cheese’s textural properties, contributing to a denser and more cohesive structure [12]. Homogenizing cream with UF milk improved Cheddar cheese yield, fat recovery, and meltability without affecting flavor [13]. One of the studies found that cold UF retentates to standardize milk for parmesan cheese production significantly increased cheese yield and protein recovery, with no significant differences in the pH, moisture content, proteolysis, free fatty acids, or sensory attributes [14]. The researchers found that incorporating UF retentates into Swiss cheese-making significantly reduced whey volume and increased yield without affecting ripening characteristics or sensory quality [15]. Despite its advantages, a primary challenge in UF is membrane fouling, predominantly caused by the formation of a casein micelle layer on the membrane surface. However, this can be reduced by processing parameter changes [16,17]. The matter is complicated when whole milk (WM) is used for UF processing, leading to membrane fouling.

The increasing interest in the UF of milk is mainly due to its ability to concentrate skim milk, which can then be used to produce specific soft cheeses with improved yield and desirable qualities [18]. Early research demonstrated the technical feasibility of WM UF at the laboratory level, providing a foundation for its potential application in dairy processing. A study investigated the impact of the UF process at two processing temperatures (7 °C and 50 °C) on processing performance and the physical, chemical, and sensory properties of protein beverages. UF at 50 °C significantly improved processing efficiency, nearly doubling the flux rate compared to at 7 °C. The UF temperature also affected the mineral partitioning and pH of the resulting beverages, while viscosity differences were primarily influenced by subsequent heat treatments and protein concentration [19].

Studies have demonstrated that WM concentrate can be successfully applied in the production of hard cheese, medium-fat soft cheese, and yogurt. The yield of hard cheese produced from WM concentrate was higher compared to regular whole milk, indicating further applications for UF in cheese making [20]. The potential of UF in creating milk powders for chocolate has also been studied. Increasing the protein content of WM and spray-drying results in powders with higher free-fat content and larger particle sizes. These findings offer opportunities for customized formulations to enhance chocolate production [21].

A deeper understanding of its processing mechanism is essential for advancing the UF process for WM. Studies on membrane fouling during the UF of pasteurized WM on a pilot scale revealed a significant decline in permeate flux, primarily due to early-stage fouling from protein adsorption [22]. This fouling was mainly attributed to whey proteins, particularly α-lactalbumin and β-lactoglobulin, which comprised about 95% of the foulants, with minimal casein involvement. Consequently, fouling not only reduces flux but also lowers separation efficiency and raises energy costs [23]. Various strategies have been explored to reduce fouling, including the selection of different membranes. Additionally, implementing various treatments could potentially enhance fouling control and make the filtration system more efficient. Homogenization is a process that reduces particle size and creates a uniform dispersion, which can enhance the efficiency of UF in various applications and prevent membrane fouling. Studies on the effect of ultra-high pressure 200–300 MPa homogenization on milk processing have observed a significant improvement in UF efficiency due to enhanced fat dispersion, increased protein retention, and modified casein micelle interactions [5]. Furthermore, homogenization at high temperatures significantly enhances UF by increasing flux rates and reducing processing time.

Most milk fat is liquid at temperatures above 42 °C. Higher temperatures during the UF process maintain milk fat in a liquid state, reducing membrane fouling by minimizing fat deposition due to decreased viscosity and increasing permeation flux. Additionally, higher temperatures reduce pumping energy by 2.3 times, improving the overall efficiency of the filtration process [24]. Research has demonstrated that UF at 50 °C achieved nearly double the flux rate compared to 7 °C, with greater protein retention and a higher Ca:P ratio in the retentate with improved mineral balance and product stability [19].

Previous studies have highlighted the advantages and challenges of the UF process, emphasizing the importance of understanding the processing parameters and the impact on filtration performance. However, limited research has explored the effects of varying temperatures and homogenization as UF treatments of WM or the resulting composition of retentate and permeate, including mineral composition. This study investigates how various milk treatments, such as homogenized or non-homogenized WM, processed at cold and hot temperatures, affect processing efficiency, composition, and minerals. Additionally, cold skim milk was also processed by UF for comparison. By analyzing key factors such as pressure, temperature, and treatment type, this research aims to provide valuable insights for optimizing the UF process, ultimately enhancing the quality and operational efficiency of dairy products. Understanding these factors can help improve membrane performance and enhance compositional knowledge, including minerals for cheese making, yogurt, and beverages, leading to better product consistency, extended membrane lifespan, and increased sustainability in dairy processing.

## 2. Materials and Methods

### 2.1. Experimental Design and Statistical Analysis

**Figure 1:**
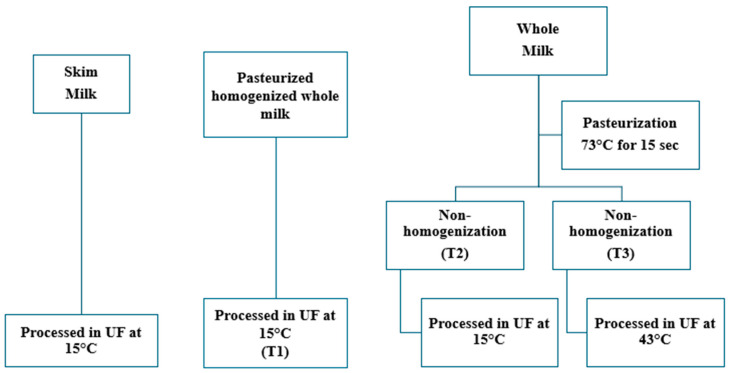
Experimental design for the different treatments during UF: SM processed in UF at 15 °C; T1 = Homogenized WM processed in UF at 15 °C; T2 = Non-homogenized WM processed in UF at 15 °C; T3 = Non-homogenized WM processed at 43 °C.

The experiments utilized commercially available pasteurized and homogenized WM (Great Value, Walmart, AZ, USA) and skim milk (Great Value, Walmart, AZ, USA). Three different lots of milk were procured over a four-week period. For the non-homogenized treatment, WM was procured on three different days from Davis Dairy Plant (South Dakota State University, Brookings, SD, USA), pasteurized (73 °C for 15 s), and then cooled. Each WM and skim milk lot served as a replicate. Hence, each experiment was replicated three times. All the lots were processed in UF as per the experimental design outlined in Figure 1. The experiment investigated the effects of varying temperatures and homogenization on WM’s filtration, processing efficiency, and composition (Figure 1). The treatments included T1: homogenized WM, processed in UF at 15 °C; T2: non-homogenized WM, processed in UF at 15 °C; T3: non-homogenized WM, processed in UF at 43 °C; and skim milk processed in UF at 15 °C. The main goal of T1 was to use homogenized milk fat (reduced fat globule size), and T2 was to investigate the effect of processing non-homogenized WM at low temperature. In contrast, for T3, the goal was to keep fat in a liquid condition by processing the milk in UF at 43 °C. The initial milk, concentrates, and permeate samples were collected and analyzed for proximate composition, mineral content, and hydrodynamic diameter. The WM treatments were subjected to statistical analysis using RStudio (Version 4.4.0) at different concentrations (initial, 2X, 3X, and permeate). One-way ANOVA was performed to obtain *p*-values. The significant values were compared using Tukey’s test, and the results were considered significant at *p* < 0.05. The average flux (g/m^2^ h) and cumulative permeate removal rates (g) were calculated and compared across different treatments.

### 2.2. UF Operation

**Figure 2:**
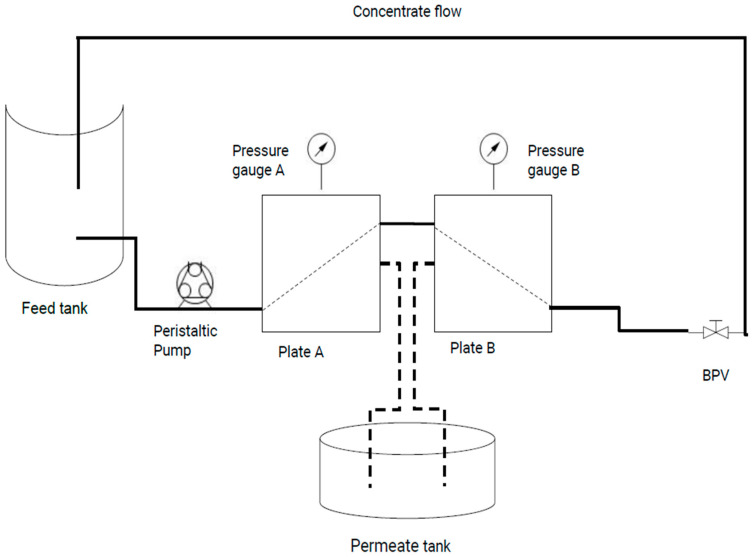
Benchtop UF unit.

The milk was subjected to the required operating temperatures for the UF processing. The experiments were conducted using an Optisep 400 filtration system (SmartFlow Technologies, Sanford, NC, USA) equipped with two 10 kDa polyethersulfone (PES) flat-sheet membranes supplied by Synder Filtration, CA, USA. The membranes, with a total surface area of 0.041 m^2^, were arranged in a series configuration. Throughout the filtration runs, a constant pressure of 50 psi was maintained. The benchtop setup (Figure 2) involved circulating milk through the system using a Masterflex L/S peristaltic pump (Model no. 7528-10, Cole Parmer, Thermo Fischer Scientific, Waltham, MA, USA). Initially, the milk flowed through plate A, where the permeate was collected. The remaining retentate was then directed to plate B for further filtration. In this series setup, the tangential flow configuration ensured the fluid moved parallel to the membrane surface, which was essential for maintaining filtration efficiency. Permeate from both plates was combined in a permeate collection tank for subsequent analysis. A back-pressure valve positioned after plate B was used to maintain a pressure of 50 psi throughout the filtration process. The temperature was maintained using a temperature-controlled water bath, with either ice water at 15 ± 1 °C or warm water at 43 ± 1 °C. In each experiment, 1200 g of milk was used as the initial feed. During filtration, 800 g permeate was removed to achieve a target concentration factor of 3×. The concentration factor was calculated by dividing the initial feed mass by the final retentate mass, where the final retentate mass is the amount of milk remaining after the removal of 800 g of permeate. Each treatment was conducted using three independent production lots, and each lot was processed once per treatment. Between trials, the entire UF system was cleaned using a warm soap solution, followed by rinsing with potable water. The membrane sheets were then replaced with new 10 kDa membranes for each trial.

#### Processing Performance

To evaluate the membrane’s performance, the permeate flux rate was calculated based on the mass of permeate collected. The permeate flux (J) is defined as the mass of permeate per unit membrane area and time, and was calculated using the formula:

J = M/A × t

where J is the permeate flux (g/m^2^·h), M is the mass of permeate collected (g), A is the membrane area (m^2^ for each membrane), and t is the time (hr) over which the permeate was collected [7]. Calculating the permeate flux rate at regular intervals provided insights into changes in membrane performance throughout the filtration process.

Throughout each experiment, key variables were measured to monitor system performance, including the mass of permeate and inlet and outlet pressures at Plate A and Plate B, to observe pressure differentials across the membranes. Additionally, the feed temperature was carefully controlled depending on the treatments. For SM, T1, and T2, ice water was used to maintain the temperature at 15 °C, while for T3, unhomogenized milk was kept at a constant temperature of 43 °C using a controlled water bath. The feed vessel was covered with aluminum foil, leaving a hole for the feed line, and placed in the water bath. Additionally, the water bath itself was covered with a lid to minimize heat loss. Temperature was monitored at regular intervals using a digital thermometer to ensure uniform thermal distribution and minimize fluctuations throughout the filtration process. The cumulative permeate removal rates were calculated by dividing the total mass of permeate collected by the time.

### 2.3. Proximate Chemical Analysis

The initial milk of SM, T1, T2, and T3, retentate (2X, 3X, final), and permeate samples was collected and immediately cooled to 4 °C. Duplicate analysis for total solids (TSs), fat %, crude protein % (CP), NPN, and non-casein nitrogen % (NCN) content was determined. The TS content was determined using forced-air oven drying at 100 °C for 5–6 h [25]. The fat percentage was measured by the Mojonnier ether extraction method [25]. CP%, NPN%, and NCN% were measured using standard procedures using the Kjeldahl method [25]. The true protein (TP) was calculated by subtracting NPN from CP; casein nitrogen (CN) was calculated by subtracting the NCN from CP and multiplying by 6.38; and serum protein (SP) was calculated by subtracting CN from TP. The ratios of CN/CP, SP/CP, NPN/CP, CN/TP, SP/TP, and NPN/TP were used to assess the distribution of protein fractions. The ash was determined by placing the samples in a muffle furnace at 550 °C after evaporating residual moisture over a hot plate [25]. Samples were ashed overnight in a muffle furnace, cooled and digested with concentrated nitric acid, and heated to dryness. The ash was then dissolved in dilute hydrochloric acid and nitric acid. The supernatant was analyzed for major minerals (Ca, K, P, Mg, Na, and Zn) and trace minerals (Cu, S. Fe, and Mn) through atomic absorption spectroscopy (AAS) [25].

### 2.4. Hydrodynamic Diameter

The mean values of hydrodynamic diameter (µm) of initial, 2X, 3X, Final, and permeate were measured using a particle size analyzer (Litesizer 500, Anton Paar, Ashland, VA, USA) at 27 °C, according to the method described by Olson et al. [26]. Further, 1 mL of samples from different treatments was diluted 1:1000 with distilled water to achieve a transmittance above 85%. The cuvette (Omega, Anton Paar, Ashland, VA, USA), designed to determine particle size, was filled with a diluted sample. The hydrodynamic diameter (µm) values were measured in duplicates.

## 3. Results and Discussions

### 3.1. Flux Rate and Permeate Removal

**Figure 3:**
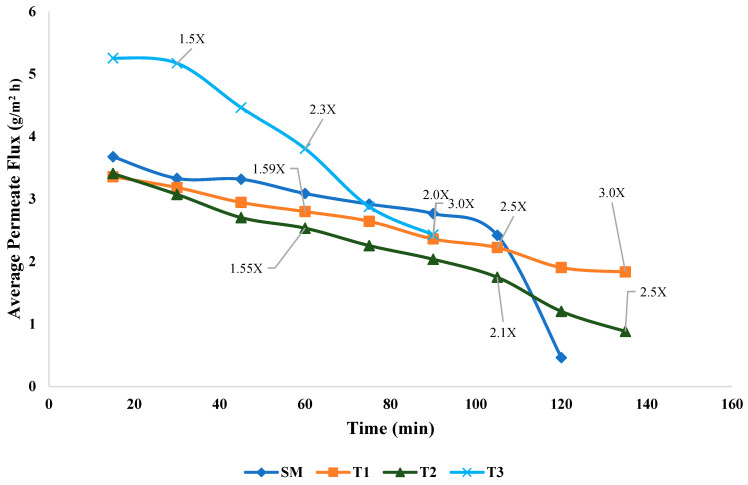
Average permeate flux (g/m^2^ h): SM processed in UF at 15 °C; T1 = Homogenized WM processed in UF at 15 °C; T2 = Non-homogenized WM processed in UF at 15 °C; T3 = Non-homogenized WM processed at 43 °C (n = 3).

The permeate flux (g/m^2^ h) across different treatments, SM, T1, T2, and T3, as in Figure 3, showed a progressive decline as filtration progressed, with noticeable variations among treatments. SM demonstrated the highest initial permeate flux rate compared to T1 and T2 treatments, which steadily declined after 100 min, indicating fouling or concentration polarization. The higher SM initial permeate flux was consistent, and filtration occurred at a faster rate. This could be attributed to the absence of fat, which significantly reduced the potential for membrane fouling caused by fat globules [27]. At the initial phase of UF, both T1 and T2 exhibited similar permeate flux, starting around 3 g/m^2^ h, because both treatments had similar initial compositions, with the main difference being the homogenization of fat globules in T1. By the final stage, T1 reached 3× concentration around 140 min, indicating that the homogenization had broken down the fat globules into smaller particles, which reduced the rate of pore blockage and allowed for more consistent filtration performance, leading to a higher concentration (Figure 3). In contrast, the permeate flux of T2 declined more steeply over time and only reached 2.5× concentration by 140 min. This was likely due to the presence of larger fat globules in unhomogenized milk, which caused faster fouling as the membrane pores clogged more easily, reducing overall filtration efficiency. Membrane fouling, characterized by the deposition and accumulation of particles on the membrane surface and within its pores, leads to an irreversible decline in flux over time, as observed in the case of T2, where increased hydrodynamic resistance and localized solute concentration due to concentration polarization further contributed to the decline in filtration performance. This irreversible decline in flux referred to a persistent drop in the filtration rate that could not recover, even if the process was stopped or the system was rinsed. It typically occurs when substances, such as fat globules or proteins, become trapped deep within the membrane pores and form a tightly packed layer that is difficult to remove. In T2, the milk was not homogenized; the larger fat globules likely clumped together and built up on the membrane more quickly, creating a dense layer that blocked the flow and made filtration less efficient over time. The accumulation of solutes near the membrane surface created an additional barrier to flow, compounding the effects of fouling and further limiting the filtration efficiency [1].

The T3 treatment exhibited a distinct initial permeate flux pattern compared to the other treatments. Initially, the permeate flux started at a significantly higher rate due to heat treatment at 43 °C, which reduced the milk’s viscosity and enhanced permeate flow. This increase in permeate flux enabled faster filtration, effectively reducing the filtration time by nearly half compared to the other treatments. The filtration for T3 was terminated upon reaching a 3× concentration factor rather than due to a decline in permeate flux. Although at 43 °C the overall micelle size remains constant, the internal structure induces some modifications that alter its hydration properties and affect the permeability of serum proteins and minerals through the membrane [28]. Shifts in mineral equilibrium at 43 °C may have caused calcium and phosphate to move from micelles into the solution compared to T1, promoting mineral–casein complex formation near the membrane and further contributing to fouling. Despite these challenges, the T3 treatment achieved a 3× concentration within approximately 80 min, aided by its initially elevated permeate flux. This suggests that operational conditions and physicochemical changes, rather than conventional fouling, primarily influenced the permeate flux behavior in T3 treatment. A previous study demonstrated that the UF of SM at 50 °C nearly doubles the permeate flux compared to cold UF [19].

**Figure 4:**
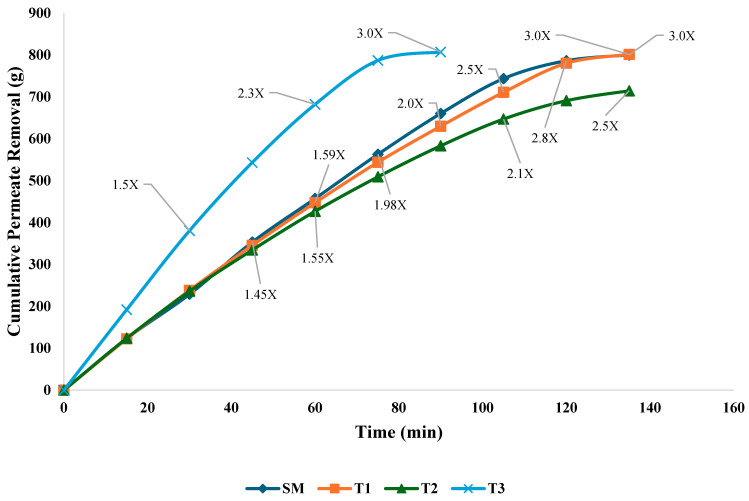
Permeate removal (g): SM processed in UF at 15 °C; T1 = Homogenized WM processed in UF at 15 °C; T2 = Non-homogenized WM processed in UF at 15 °C; T3 = Non-homogenized WM processed at 43 °C (n = 3).

The cumulative permeate removal for different milk treatments is shown in Figure 4. The permeate removal generally increased with time, but the rate differed across the various treatments. SM and T1 followed a very similar path in cumulative permeate removal, exhibiting steady permeate removal and eventually reaching 3X concentration around 140 min. T3 initially exhibited the highest permeate removal rate, reaching 1.5X concentration within 40 min, which could be attributed to the effects of heat treatment. Heat treatment likely reduced viscosity and enhanced permeate flow, facilitating faster filtration in the initial phase. Supporting this observation, previous studies examined the influence of processing temperatures of 10 °C, 30 °C, and 50 °C on flux behavior during the UF of SM and reported that permeate viscosity significantly decreased at 50 °C (0.63 cP), which resulted in an increased initial flux compared to lower temperatures. In contrast, at 10 °C, the higher viscosity (1.49 cP) resulted in a reduced flux [29]. The breakdown of larger protein structures into smaller aggregates or complexes during heat treatment contributes to a more homogenous and less viscous liquid, facilitating permeate flow [30].

Both T1 and T2 followed similar trends in permeate removal during the initial stages. However, T1 demonstrated better performance compared to T2, reaching 3X concentration by 140 min. This difference can be attributed to the homogenization process in T1, which reduced the size of fat globules and helped mitigate fouling [31]. Homogenization reduces the size of fat globules, creating a more uniform emulsion that alters the physical characteristics of the fat–protein matrix during ultrafiltration (UF). Based on our findings, we hypothesize that homogenization in T1 resulted in a more evenly dispersed fat phase, leading to the formation of a less compact and more porous deposit layer on the membrane, which may have mitigated membrane fouling and gel polarization compared to T2. In a previous study using homogenized whole milk, extrapolation of flux–concentration data indicated that milk could be concentrated up to a factor of 3.3, primarily due to increasing resistance from the fat–protein concentration boundary layer and gel polarization [19]. However, that study did not compare the specific effects of homogenized versus non-homogenized milk on membrane fouling behavior. In contrast, the T2 treatment only reached 2.5× concentration by the end of the process. The presence of larger fat globules in non-homogenized milk causes a higher tendency for fouling, resulting in reduced filtration efficiency. This fouling leads to a buildup of solids on the membrane surface, forming a secondary membrane layer that alters the net sieving and transport properties of the system, ultimately decreasing the permeate rate [1]. UF processing temperature and homogenization influence UF efficiency. Heat treatment enhances initial flux and reduces viscosity, thereby facilitating faster filtration. Homogenization, on the other hand, improves membrane performance by reducing fat globule size, minimizing pore blockage, and leading to higher concentration levels.

### 3.2. Initial Milk Composition

The mean compositional values for SM and the three treatments (T1, T2, T3) are presented in Table 1, with statistical analysis conducted only for the three WM treatments. The results show that SM had the lowest TS and fat, as removing cream from skim milk significantly reduces the fat and TS contents compared to WM samples. The initial WM samples from different treatments showed no significant difference in fat and TS%. The ash, CP, TP, and CN content showed significant (*p* < 0.05) changes, with only minimal variations observed, representing week-to-week fluctuations in the raw milk supply. In contrast, differences in NCN, NPN, and SP content were nonsignificant. In the present study, the average CN/CP and CN/TP ratios of SM were 76.24% and 80.63%, respectively. Similar values have been reported in other studies [8,32,33]. However, the initial WM with different treatments showed higher values for the CN/CP and CN/TP ratios because of the higher CN content present in the milk. Comparable compositional values and ratios were also documented in the WM study conducted by others [34].

### 3.3. Retentate Composition at 2X, 3X, and Final Concentration Levels

The mean composition of SM and the statistical analysis of three treatments of retentate at the 2X concentration level are shown in Table 2. There was no significant difference in the TS and fat content among the treatments, with T2 showing higher values. At 2X concentration, significant increases were observed in the protein fraction values for T1 compared to the other WM treatments; however, the values were lower than those for SM. The CP of T1 showed significantly *(p* < 0.05) higher values compared to T2 and T3. The NCN content of T1 was also significantly *(p* < 0.05) different from the T2 treatment.

The mean composition, protein fractions, and protein ratios of SM, T1, and T3 at 3× retentate are given in Table 3. The T2 is not shown in Table 3, as it presented difficulties in achieving a 3× concentration (Figure 2) during UF because the cold UF processing impacted the membrane flow with higher viscosity and promoted aggregation, limiting concentration efficiency. The fat content of T2 was higher than that of T1 due to filtration conducted at 43 °C, which may keep the fat in a liquid phase and result in less fat loss in the permeate. Homogenization reduces the size of fat globules and creates a more uniform emulsion, which alters the physical characteristics of the fat–protein matrix during UF. Based on our findings, we hypothesize that homogenization in T1 resulted in a more evenly dispersed fat phase, leading to the formation of a less compact and more porous deposit layer on the membrane, which may have mitigated membrane fouling and gel polarization compared to T2. The ash content was significantly higher (*p* < 0.05) in T1, which may indicate that mineral concentration variations were influenced by homogenization. According to Meena et al. (2016), the authors investigated the effects of UF concentration, homogenization, and stabilizing salts on the heat stability, zeta potential, and rheological properties of cow skim milk UF retentate [35]. They found that the heat coagulation time (HCT) of UF retentate significantly decreased after homogenization, which was attributed to structural modifications of milk proteins and altered protein–mineral interactions, particularly with calcium [35]. The homogenization process induces shearing and cavitation effects, resulting in changes to the surface characteristics of proteins and disrupting the natural equilibrium between casein and calcium phosphate. Additionally, homogenization and increased calcium concentration were found to significantly reduce the zeta potential of casein micelles from −0.598 mV to −1.007 mV, indicating a decrease in colloidal stability and a higher propensity for heat-induced coagulation [35]. T1 achieved significantly (*p* < 0.05) higher CN levels than T3 during UF, which may be due to the lower fat observed in T1. No significant difference was observed in other protein ratios.

Table 4 shows the compositional differences across the different milk types at the final concentration during UF. The TS content at the final concentration was highest in T1, with the highest retention of solid components. The fat content varied significantly among the three treatments. In T1, the fat globules are broken into smaller, uniformly distributed particles, resulting in a more stable emulsion. This stability enables a better separation of fat and minimizes the likelihood of fat globules near the membrane, resulting in lower fouling (Figure 3). The ash content in T1 was significantly higher, indicating that homogenized milk retains a slightly higher mineral content than T3, whereas the T2 treatment showed the lowest ash content among WM treatments due to its limited concentration of only 2.5×. T1 achieved significantly (*p* < 0.05) higher CP, CN, and SP values and was close to the SM. There was no significant difference in protein ratios. However, protein fraction ratios help to understand the protein functionality. The different treatment results can be useful for future research exploring protein composition and functionality in dairy products. It is important to note the final concentration ratio to understand the length of the process before fouling occurs.

### 3.4. Permeate Composition Analysis

The composite permeate composition across different treatments is shown in Table 5. The variation in the permeate composition was observed mainly due to different treatments, which altered the net sieving and transportation properties of the membrane. SM permeate values were similar to those reported in a study by Salunke et al. [9]. The TS was lowest in T1 at 3.32% and highest in T3 at 4.37%. The fat content in the permeate sample was lowest in T3, but the differences were too low to be significant when compared to the T1 and T2 samples. The ash content varied across the samples, with T2 having a significantly (*p* < 0.05) higher value. The CP, NPN, and NCN were also higher in T3 due to a lack of homogenization combined with heat treatment, which promotes protein breakdown and increases the passage of nitrogen fractions into the permeate [20]. There was no significant difference in TP, CN, SP content, or protein ratios.

### 3.5. Retentate and Permeate Mineral Analysis

The mean mineral content (mg/100 g), including Ca, K, P, Na, Mg, and Zn, of SM at the initial, 2×, 3×, and final concentration levels is presented in Table 6 for reference and comparison. The mineral content of the whole milk treatments (T1, T2, T3) at initial, 2×, and 3× concentration levels are provided in Table 7, while the final concentration and permeate mineral composition of whole milk treatments (T1, T2, T3) are given in Table 8. These results highlight significant differences across treatments and concentration levels. The means of trace mineral content (mg/100 g) obtained are given in Table 9.

All the WM treatments (T1, T2, T3) (Table 7) and SM (Table 6) showed similar Ca levels at the initial stage of UF, with no significant differences, and with SM having slightly higher Ca. In the 2X concentration, the Ca content was significantly (*p* < 0.05) higher in T1, while T2 and T3 showed significantly (*p* < 0.05) lower values. This trend persisted during 3X concentration, but T2 did not reach the expected concentration level. The significantly lower Ca content in T2 final concentrate can be partially attributed to its significantly lowest CN content (Table 4) among all treatments, resulting in a greater loss of Ca in permeate (Table 8), as most Ca is present in milk in the form of colloidal calcium phosphate (CCP) bound to casein micelles. During UF, the high fat content in T2 leads to fat globule accumulation on the membrane surface, forming a secondary hydrophobic layer that hinders casein micelle transmission toward the membrane. This phenomenon may be the partitioning of Ca between retentate and permeate [36]. Heat treatment in T3 reduced Ca solubility by promoting protein aggregation and rearrangement, limiting its concentration in the final product as compared to T1 (Table 8) [37]. There was no significant difference in the Ca content in permeate among the treatments, although the values were slightly higher in T2, followed by T3 and T1 due to two different treatments (Table 8). The Ca values observed in the UF permeate for SM, T1, and T3 were consistent, which suggests that the UF effectively retained Ca in the concentrate, with minimal removal into permeate.

The initial T2 milk had higher K levels compared to other treatments (Table 7). However, the final concentration of T2 had a significantly (*p* < 0.05) lower K level (Table 8). This reduction was possibly due to the larger fat globules in non-homogenized milk, which allowed more free K in serum phase [38]. This resulted in a significantly (*p* < 0.05) higher K content in the T2 permeate (Table 8). On the other hand, T1 was subjected to homogenization, which created smaller fat globules and modified the fat–protein interface, potentially reducing the amount of free K available for filtration [39]. This resulted in a lower K content in the permeate (Table 8).

Table 7 shows that T2 consistently had a lower P content compared to other treatments, particularly at the higher concentration stage, 2X (Table 7), and final concentration (Table 8). P in milk is largely associated with casein micelles and exists in colloidal form. During homogenization, the fat globules reduced in size, which might have increased the exposure of P bound to casein micelles, thereby retaining more P in the concentrated phase [40,41]. On the other hand, T2 may have retained less P in the retentate due to lower interaction between P and the fat–protein matrix. The larger fat globules in non-homogenized milk may have reduced P availability in the retentate. The P content of T1 in permeate was significantly (*p* < 0.05) lower because most P is retained in the retentate with casein micelles. However, there was a significant (*p* < 0.05) difference in permeate from non-homogenized treatments, which could indicate a weaker retention of P in the non-homogenized milk, supporting the higher P content in the permeate (Table 8).

All milk treatments (Table 7) and SM (Table 6) showed similar Na levels at the initial stage of UF. During 2× and 3× concentrations, Na content was highest in T1, while T2 and T3 had significantly lower (*p* < 0.05) values (Table 7). T2 exhibited the lowest Na content in the final concentration. This pattern could be due to the monovalent nature of sodium, which is less likely to interact with proteins and fat compared to divalent minerals like Ca. However, osmotic forces and charge interactions still affected Na in the retentate [42]. Additionally, membrane selection and polarization affect the membrane surface to trap both types of ions [43].

The Mg content of the T1 treatment was significantly (*p* < 0.05) higher, particularly at the 3× (Table 7) and final concentration level (Table 8). Mg is mostly bound to casein micelles or present as free ions in the serum phase. Homogenization affects the fat–protein matrix by reducing the size of fat globules, potentially allowing better retention within the casein micelle [44]. The T3 treatment exhibited lower Mg content in the 3× concentration (Table 7) and final stage (Table 8), possibly due to heat-induced changes, which can alter the mineral-binding capacity [45]. We hypothesize that T3 had a lower Mg content compared to T1, possibly due to mild conformational changes in proteins, which influence micelle dynamics and affect the distribution of Mg. Additionally, the absence of homogenization may have contributed to a reduced interaction of Mg with the protein matrix, impacting its retention during UF. The larger fat globules in the non-homogenized samples may have reduced the interaction of Mg with the protein matrix, leading to lower retention and significantly (*p* < 0.05) higher Mg content in the permeate (Table 8).

Zinc (Zn), a trace mineral present in milk, showed some interesting patterns across the different treatments and concentration stages. The T1 and T3 treatments had relatively higher Zn retention compared to T2 in the final concentration stages (Table 8). Zn is primarily associated with proteins in milk, mostly with casein micelles and, to a lesser extent, with whey proteins [39]. Homogenization, by reducing fat globule size and altering the fat–protein interface, could increase Zn retention by enhancing its association with casein micelles, as seen in the higher levels in the T1 sample (Table 8) [38]. The T3 showed a similar Zn retention to T1 at the final concentration stage. This may be due to the native interactions between Zn ions and milk proteins, particularly casein micelles. Zn exhibits a strong affinity for phosphate groups and negatively charged surface amino acids. Even at 43 °C, molecular mobility can be altered through protein structural changes. Overall, the retention pattern indicates that processing treatments such as homogenization and heat can influence its distribution in concentrated milk products.

The details of the trace elements are given in Table 9. The trace elements were concentrated according to the ratio of concentration, and as the concentration in retentate increased, the mineral amount increased. The losses in permeate were similar in all treatments. The increase in concentration of these minerals in retentate or permeate (if converted to serum protein or whey-based ingredients) can affect the functionality of the products in which they are used.

### 3.6. Hydrodynamic Diameter

**Figure 5:**
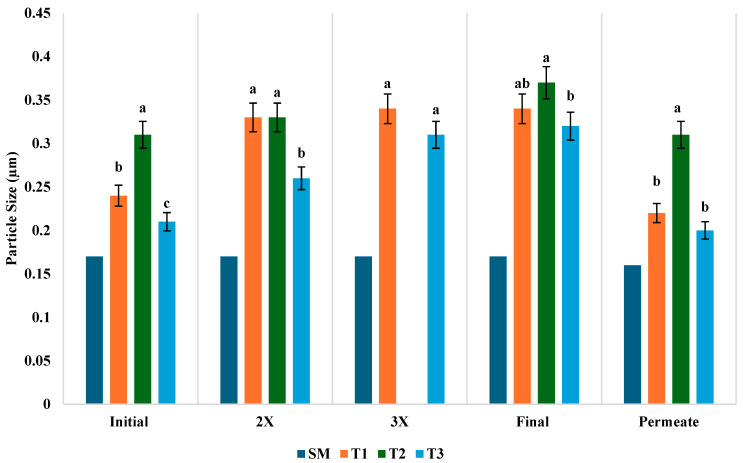
Hydrodynamic diameter (µm) SM processed in UF at 15 °C; T1 = Homogenized WM processed in UF at 15 °C; T2 = Non-homogenized WM processed in UF at 15 °C; T3 = Non-homogenized WM processed at 43 °C (n = 3). Bars that do not share common letters are considered significantly different (*p* < 0.05).

The hydrodynamic diameter represents the effective size of particles or molecules in a hydrated state, which is critical for assessing their stability, aggregation, and behavior in various fluid systems, including colloidal suspensions [46]. The changes in particle size distribution (µm) for SM and three different treatments (T1, T2, T3) across different stages of concentration (initial, 2×, 3×, final) and permeate obtained during the UF are shown in Figure 5. The hydrodynamic diameter differences among types of milk and treatments may be due to processing conditions (cream separation, preheating, homogenization, and heat treatment during UF). At the initial stages of UF, the particle size of SM had the smallest particle size of 0.17 µm due to the absence of fat. Similar results were found in a study conducted by Jhanwar and Ward [47]. However, in the treatments, T2 showed the largest particle size of 0.31 µm, followed by T1 at 0.24 µm and T3 at 0.21 µm. The T1 value is lower than the T2 treatment because of the homogenization pressure [48]; however, it is slightly higher than T3 treated milk, which may be due to the aggregation of small fat particles during the storage of homogenized milk [49,50].

As the milk samples were concentrated to 2×, the particle size increased in whole milk samples, indicating a slight aggregation of fat globules and proteins. The SM particle size remained constant at 0.17 µm, and was not affected during this stage. At the 3× concentration stage, the particle size of T3 increased slightly. At the final concentration, T2 showed a significantly (*p* < 0.05) larger particle size, followed by T3 and T1, whereas SM remained constant at 0.17 µm throughout all the concentration stages. The final concentration of all the treatments showed higher results as compared to the initial milk. The T3 sample showed higher particle values, which may be due to whey protein rearrangement which varied with protein concentration and heat treatment temperature, as compared to the initial, 2×, and 3× concentrations [51]. The permeate of all the samples collected during UF contained smaller particles than the retentate, as the filtration membrane retains larger particles. SM permeate had the smallest particle size at 0.16 µm. In the other three treatments, T2 showed significantly (*p* < 0.05) higher particle distributions than T1 and T3, which can be attributed to the absence of homogenization, lower temperature, and incomplete concentration during UF. Even though there was a slight change in particle size in the retentate, it did not affect the UF processing operation.

## 4. Conclusions

This study highlighted the influence of homogenization and temperature on UF efficiency and the compositional properties of whole milk retentate and permeate. Homogenization in T1 improved filtration performance by reducing fat globule size, minimizing membrane fouling, and enhancing protein and mineral retention. This treatment is well-suited for the production of value-added dairy products, such as high-protein yogurts, cheese milk bases, and fortified beverages. Heat treatment in T3 initially increased flux by reducing viscosity but induced physicochemical changes that impacted mineral distribution and protein aggregation, changing the filtration process. In contrast, non-homogenized, cold-processed WM (T2) exhibited higher fouling tendencies due to larger fat globules, resulting in a lower concentration efficiency. The study shows that modifying fat using homogenization (to reduce the size of fat) and higher temperatures (to keep fat in a melted or liquid condition) helps in UF processing. These findings provide valuable insights for optimizing UF processes in WM dairy applications, particularly for achieving targeted compositions in concentrated milk products.

## Figures and Tables

**Table 1:** Mean (n = 3) composition (% by weight) of skim milk and whole milk during UF.

Treatment	TS	Fat	Ash	CP	NCN	NPN	TP	CN	SP	CN/CP	SP/CP	NPN/CP	CN/TP	SP/TP	NPN/TP
**SM** **SD**	9.64 0.12	0.33 0.03	0.68 0.01	3.45 0.04	0.82 0.03	0.19 0.01	3.27 0.03	2.63 0.06	0.63 0.03	76.24 1.03	18.31 1.00	5.44 0.03	80.63 1.07	19.37 1.07	5.76 0.03
**T1** **SD**	12.68 0.05	3.38 0.02	0.66 ^a^ 0.01	3.41 ^b^ 0.01	0.67 0.02	0.22 0.03	3.20 ^b^ 0.03	2.74 ^b^ 0.01	0.46 0.02	80.32 0.42	13.38 0.54	6.30 ^a^ 0.95	85.73 0.43	14.27 0.43	6.73 ^a^ 1.10
**T2** **SD**	12.35 0.72	2.65 0.77	0.67 ^a^ 0.04	3.41 ^b^ 0.05	0.65 0.02	0.18 0.01	3.23 ^b^ 0.04	2.76 ^b^ 0.05	0.47 0.02	80.99 0.53	13.68 0.64	5.33 ^ab^ 0.15	85.55 0.66	14.45 0.66	5.63 ^ab^ 0.17
**T3** **SD**	12.36 0.74	2.70 0.78	0.52 ^b^ 0.06	3.54 ^a^ 0.06	0.65 0.07	0.17 0.02	3.37 ^a^ 0.05	2.89 ^a^ 0.04	0.48 0.07	81.58 1.82	13.67 1.76	4.74 ^b^ 0.37	85.64 1.85	14.36 1.85	4.98 ^b^ 0.41

SM processed in UF at 15 °C; T1 = Homogenized WM processed in UF at 15 °C; T2 = Non-homogenized WM processed in UF at 15 °C; T3 = Non-homogenized WM processed at 43 °C; TS = total solids; CP = total nitrogen × 6.38; NCN = non casein nitrogen × 6.38; NPN = non protein nitrogen × 6.38; TP = true protein (CP − NPN); CN (Casein) = CP − NCN; SP (serum protein) = TP − casein. Values with different letters in superscripts (a–b) within the same column differ significantly (*p* < 0.05).

**Table 2:** Mean (n = 3) composition (% by weight) of different treatments at 2× concentration (retentate) during UF.

Treatment	TS	Fat	Ash	CP	NCN	NPN	TP	CN	SP	CN/CP	SP/CP	NPN/CP	CN/TP	SP/TP	NPN/TP
**SM** **SD**	14.35 0.12	0.60 0.08	1.08 0.01	8.18 0.15	1.56 0.08	0.20 0.01	7.97 0.15	6.62 0.21	1.35 0.08	80.94 1.21	16.56 1.15	2.49 0.07	83.02 1.19	16.98 1.19	2.56 0.07
**T1** **SD**	20.94 0.32	6.27 0.16	1.06 ^a^ 0.05	7.99 ^a^ 0.19	1.43 ^a^ 0.20	0.27 0.07	7.72 ^a^ 0.12	6.56 0.17	1.17 ^a^ 0.17	82.11 2.31	14.59 2.10	3.30 0.80	84.91 2.18	15.09 2.18	3.42 0.86
**T2** **SD**	21.28 0.72	8.19 0.72	0.93 ^b^ 0.01	7.00 ^b^ 0.48	0.94 ^b^ 0.21	0.23 0.01	6.76 ^b^ 0.48	6.06 0.54	0.70 ^b^ 0.22	86.56 3.37	10.12 1.12	3.32 0.24	89.53 3.50	10.47 3.50	3.43 0.25
**T3** **SD**	20.57 0.75	7.70 1.68	0.99 ^ab^ 0.04	7.01 ^b^ 0.19	1.24 ^a^ 0.08	0.29 0.10	6.72 ^b^ 0.13	5.76 0.22	0.96 ^ab^ 0.10	82.24 1.33	13.73 1.80	4.03 0.77	85.70 1.79	14.30 1.79	4.20 0.83

SM processed in UF at 15 °C; T1 = Homogenized WM processed in UF at 15 °C; T2 = Non-homogenized WM processed in UF at 15 °C; T3 = Non-homogenized WM processed at 43 °C; TS = total solids; CP = total nitrogen × 6.38; NCN = non casein nitrogen × 6.38; NPN = non protein nitrogen × 6.38; TP = true protein (CP − NPN); CN (casein) = CP − NCN; SP (serum protein) = TP − casein. Values with different letters in superscripts (a–b) within the same column differ significantly (*p* < 0.05).

**Table 3:** Mean (n = 3) composition (% by weight) of different treatments at 3× concentration (retentate) in during UF.

Treatment	TS	Fat	Ash	CP	NCN	NPN	TP	CN	SP	CN/CP	SP/CP	NPN/CP	CN/TP	SP/TP	NPN/TP
**SM** **SD**	18.08 0.28	0.91 0.03	1.29 0.03	11.45 0.05	2.04 0.04	0.22 0.01	11.24 0.06	9.41 0.02	1.82 0.04	82.20 0.24	15.91 0.28	1.89 0.06	83.78 0.28	16.22 0.28	1.92 0.05
**T1** **SD**	29.21 2.35	8.15 ^b^ 0.29	1.45 ^a^ 0.02	11.29 ^a^ 0.32	2.39 0.69	0.32 0.08	10.97 ^a^ 0.69	8.90 ^a^ 0.43	2.07 0.61	78.92 3.50	18.27 2.87	2.81 0.64	81.18 3.14	18.82 3.01	2.89 0.68
**T3** **SD**	27.77 1.26	12.13 ^a^ 1.22	1.27 ^b^ 0.11	9.51 ^b^ 0.55	1.69 0.29	0.38 0.08	9.13 ^b^ 0.59	7.82 ^b^ 0.28	1.31 0.31	82.3 2.09	13.72 2.53	3.98 0.98	85.72 2.55	14.28 2.55	4.16 1.10

SM processed in UF at 15 °C; T1 = Homogenized WM processed in UF at 15 °C; T3 = Non-homogenized WM processed at 43 °C; TS = total solids; CP = total nitrogen × 6.38; NCN = non casein nitrogen × 6.38; NPN = non protein nitrogen × 6.38; TP = true protein (CP − NPN); CN (casein) = CP − NCN; SP (serum protein) = TP − casein. Values with different letters in superscripts (a–b) within the same column differ significantly (*p* < 0.05).

**Table 4:** Mean (n = 3) composition (% by weight) of different treatments at final concentration (retentate) during UF.

Treatment	TS	Fat	Ash	CP	NCN	NPN	TP	CN	SP	CN/CP	SP/CP	NPN/CP	CN/TP	SP/TP	NPN/TP
**SM** **SD**	18.93 0.35	0.96 0.03	1.46 0.01	12.60 0.15	2.23 0.02	0.25 0.01	12.35 0.15	10.36 0.14	1.98 0.02	82.28 0.17	15.72 0.11	2.00 0.08	83.96 0.12	16.04 0.12	2.04 0.08
**T1** **SD**	29.91 1.64	10.90 0.48	1.54 ^a^ 0.03	12.47 ^a^ 0.17	2.56 ^a^ 0.42	0.36 0.11	12.11 ^a^ 0.08	9.92 ^a^ 0.26	2.19 ^a^ 0.32	79.53 3.10	17.57 2.31	2.89 0.89	81.89 2.52	18.11 2.52	2.98 0.90
**T2** **SD**	28.41 1.37	12.35 1.58	1.18 ^b^ 0.09	8.70 ^b^ 0.94	1.35 ^b^ 0.15	0.32 0.04	8.39 ^c^ 0.92	7.36 ^b^ 1.10	1.03 ^b^ 0.17	84.27 3.56	12.08 3.38	3.65 0.39	87.46 3.54	12.54 3.54	3.79 0.42
**T3** **SD**	28.15 0.94	12.76 1.29	1.47 ^a^ 0.07	10.91 ^a^ 0.55	2.07 ^a^ 0.09	0.44 0.10	10.47 ^b^ 0.50	8.84 ^ab^ 0.52	1.63 ^a^ 0.16	81.04 0.95	14.97 1.78	3.99 0.85	84.41 1.71	15.59 1.71	4.16 0.92

SM processed in UF at 15 °C; T1 = Homogenized WM processed in UF at 15 °C; T2 = Non-homogenized WM processed in UF at 15 °C; T3 = Non-homogenized WM processed at 43 °C; TS = total solids; CP = total nitrogen × 6.38; NCN = non casein nitrogen × 6.38; NPN = non protein nitrogen × 6.38; TP = true protein (CP − NPN); CN (casein) = CP − NCN; SP (serum protein) = TP − casein. Values with different letters in superscripts (a–c) within the same column differ significantly (*p* < 0.05).

**Table 5:** Mean (n = 3) composition (% by weight) of different treatments in permeate during UF.

Treatment	TS	Fat	Ash	CP	NCN	NPN	TP	CN	SP	CN/CP	SP/CP	NPN/CP	CN/TP	SP/TP	NPN/TP
**SM** **SD**	3.44 0.60	0.05 0.03	0.09 0.01	0.13 0.01	0.12 0.01	0.11 0.01	0.03 0.01	0.02 0.01	0.01 0.01	14.16 0.58	6.69 1.23	79.15 1.59	68.21 2.91	31.79 2.91	384.32 35.50
**T1** **SD**	3.32 0.59	0.33 0.10	0.17 ^b^ 0.03	0.17 ^b^ 0.01	0.14 ^b^ 0.01	0.12 ^b^ 0.01	0.04 0.01	0.02 0.01	0.01 0.01	18.17 3.52	8.66 1.24	73.17 3.69	66.63 1.84	33.37 1.84	291.54 8.20
**T2** **SD**	4.10 1.10	0.33 0.32	0.36 ^a^ 0.03	0.16 ^b^ 0.01	0.13 ^b^ 0.01	0.12 ^b^ 0.01	0.04 0.01	0.03 0.01	0.02 0.01	16.67 4.95	9.25 0.99	74.08 3.96	63.16 3.36	36.84 2.36	294.34 5.80
**T3** **SD**	4.37 1.04	0.24 0.14	0.18 ^b^ 0.01	0.24 ^a^ 0.01	0.22 ^a^ 0.01	0.19 ^a^ 0.01	0.05 0.01	0.02 0.01	0.02 0.01	10.82 3.36	8.92 0.82	80.25 3.61	54.06 2.58	42.94 2.58	418.47 8.60

SM processed in UF at 15 °C; T1 = Homogenized WM processed in UF at 15 °C; T2 = Non-homogenized WM processed in UF at 15 °C; T3 = Non-homogenized WM processed at 43 °C; TS = total solids; CP = total nitrogen × 6.38; NCN = non casein nitrogen × 6.38; NPN = non protein nitrogen × 6.38; TP = true protein (CP − NPN); CN (casein) = CP − NCN; SP (serum protein) = TP − casein. Values with different letters in superscripts (a–b) within the same column differ significantly (*p* < 0.05).

**Table 6:** Mineral content (mg/100 g) of skim milk at the initial level, 2×, 3×, final concentration levels, and permeate (n = 3).

Minerals	Initial	2×	3×	Final	Permeate
**Ca** **SD**	137.52 1.08	250.29 1.53	336.10 9.19	380.16 4.70	23.07 4.10
**K** **SD**	105.10 8.02	135.86 0.85	147.25 2.34	170.08 3.12	35.62 4.44
**P** **SD**	92.78 6.41	171.84 3.07	233.00 9.13	256.60 5.83	25.40 6.13
**Na** **SD**	44.61 1.73	52.80 2.19	59.92 2.94	76.73 0.52	26.80 1.99
**Mg** **SD**	11.99 0.07	18.92 0.89	23.70 1.52	21.72 3.90	6.84 2.84
**Zn** **SD**	0.56 0.06	1.34 0.16	1.73 0.03	1.83 0.13	0.24 0.04

Ca, K, P, Na, Mg, Zn (mg/100 g) content in skim milk (SM). SM is processed in UF at 15 °C.

**Table 7:** Mineral content (mg/100 g) of whole milk treatments at the initial level, 2× concentration, 3× concentration (n = 3).

	Initial			2X			3X		
	T1	T2	T3	T1	T2	T3	T1	T2	T3
**Ca** **SD**	121.61 10.03	126.03 9.50	116.05 8.20	234.28 ^a^ 20.86	203.12 ^b^ 1.93	213.60 ^ab^ 4.00	353.20 ^a^ 19.10	-	281.70 ^b^ 28.34
**K** **SD**	87.32 ^b^ 5.36	120.98 ^a^ 4.55	106.14 ^a^ 9.96	94.87 ^b^ 2.67	101.99 ^ab^ 6.94	110.50 ^a^ 3.93	113.01 9.60	-	112.60 4.90
**P** **SD**	88.53 12.99	96.95 5.25	83.03 2.43	152.73 ^a^ 6.90	117.60 ^b^ 7.96	135.27 ^ab^ 8.60	223.90 8.66	-	206.01 9.74
**Na** **SD**	45.80 2.44	45.77 1.40	47.13 1.12	54.91 ^a^ 5.76	39.41 ^b^ 2.70	48.73 ^ab^ 3.81	65.25 ^a^ 4.19	-	46.87 ^b^ 4.43
**Mg** **SD**	12.49 1.23	11.10 0.52	9.94 1.33	17.96 1.61	15.67 0.21	16.20 0.57	23.32 ^a^ 1.41	-	19.40 ^b^ 0.86
**Zn** **SD**	0.45 ^b^ 0.04	0.55 ^a^ 0.03	0.40 ^b^ 0.03	0.92 0.05	1.01 0.02	1.01 0.05	1.60 ^a^ 0.11	-	1.32 ^b^ 0.05

Ca, K, P, Na, Mg, Zn (mg/100 g) content in T1, T2, and T3 at the initial level, 2X, 3X concentration; T1 = Homogenized WM processed in UF at 15 °C; T2 = Non-homogenized WM processed in UF at 15 °C; T3 = Non-homogenized WM processed at 43 °C. Values with different letters in superscripts (a–b) within the same row and at initial, 2×, and 3× concentrations differ significantly (*p* < 0.05).

**Table 8:** Mineral content (mg/100 g) of whole milk treatments in final concentration and permeate (n = 3).

	Final			Permeate		
	T1	T2	T3	T1	T2	T3
**Ca** **SD**	373.13 ^a^ 13.35	271.70 ^c^ 5.22	328.08 ^b^ 1.22	15.63 1.30	25.76 1.80	22.04 8.00
**K** **SD**	127.72 ^a^ 4.23	111.53 ^b^ 7.10	124.28 ^a^ 1.83	23.69 ^c^ 2.31	87.70 ^a^ 11.65	47.30 ^b^ 7.01
**P** **SD**	238.57 ^a^ 3.87	163.75 ^b^ 11.44	230.74 ^a^ 8.43	16.26 ^b^ 1.90	27.51 ^a^ 4.64	20.50 ^ab^ 2.51
**Na** **SD**	77.86 ^a^ 1.74	43.71 ^c^ 2.42	55.96 ^b^ 6.24	23.85 ^b^ 2.15	29.35 ^a^ 2.55	22.89 ^b^ 0.52
**Mg** **SD**	24.21 ^a^ 1.13	18.94 ^b^ 0.70	21.11 ^ab^ 1.94	1.31 ^b^ 0.40	4.65 ^a^ 0.94	3.50 ^a^ 0.70
**Zn** **SD**	1.66 ^a^ 0.09	1.40 ^b^ 0.09	1.60 ^ab^ 0.06	0.16 ^b^ 0.01	0.40 ^a^ 0.10	0.23 ^b^ 0.03

Ca, K, P, Na, Mg, Zn (mg/100 g) content in T1, T2, and T3 at final concentration, and permeate; T1 = Homogenized WM processed in UF at 15 °C; T2 = Non-homogenized WM processed in UF at 15 °C; T3 = Non-homogenized WM processed at 43 °C. Values with different letters in superscripts (a–c) within the same row and at initial, 2×, 3×, and final concentrations differ significantly (*p* < 0.05).

**Table 9:** Trace mineral content (mg/100 g) of different treatments in initial, 2×, 3×, final concentration, and permeate (n = 3).

	**SM**				
	**Initial**	**2×**	**3×**	**Final**	**Permeate**
**Cu**	0.013 ± 0.005	0.025 ± 0.005	0.031 ± 0.004	0.036 ± 0.005	0.011 ± 0.004
**S**	7.358 ± 0.295	8.658 ± 0.263	10.36 ± 0.534	14.93 ± 0.730	5.461 ± 0.931
**Fe**	0.038 ± 0.011	0.080 ± 0.006	0.108 ± 0.007	0.123 ± 0.005	0.021 ± 0.004
**Mn**	0.000 ± 0.000	0.000 ± 0.000	0.033 ± 0.051	0.008 ± 0.004	0.00 ± 0.000
	**T1**				
	**Initial**	**2×**	**3×**	**Final**	**Permeate**
**Cu**	0.013 ± 0.005	0.021 ± 0.007	0.020 ± 0.008	0.031 ± 0.007	0.016 ± 0.005
**S**	8.176 ± 0.182	9.520 ± 0.332	10.56 ± 0.624	11.626 ± 0.286	5.133 ± 0.757
**Fe**	0.025 ± 0.005	0.053 ± 0.005	0.083 ± 0.005	0.110 ± 0.006	0.021 ± 0.004
**Mn**	0.000 ± 0.000	0.016 ± 0.040	0.050 ± 0.054	0.010 ± 0.000	0.001 ± 0.004
	**T2**				
	**Initial**	**2×**	**3×**	**Final**	**Permeate**
**Cu**	0.013 ± 0.005	0.021 ± 0.004	-	0.031 ± 0.004	0.008 ± 0.004
**S**	7.281 ± 0.441	8.393 ± 0.264	-	10.48 ± 0.548	5.133 ± 0.757
**Fe**	0.026 ± 0.008	0.058 ± 0.004	-	0.081 ± 0.007	0.026 ± 0.008
**Mn**	0.000 ± 0.000	0.000 ± 0.000	-	0.005 ± 0.005	0.001 ± 0.004
	**T3**				
	**Initial**	**2×**	**3×**	**Final**	**Permeate**
**Cu**	0.011 ± 0.004	0.015 ± 0.005	0.025 ± 0.005	0.030 ± 0.008	0.013 ± 0.005
**S**	6.088 ± 0.272	8.420 ± 0.155	9.533 ± 0.295	11.45 ± 0.279	5.133 ± 0.757
**Fe**	0.023 ± 0.005	0.055 ± 0.005	0.088 ± 0.004	0.120 ± 0.008	0.011 ± 0.004
**Mn**	0.000 ± 0.000	0.016 ± 0.040	0.033 ± 0.051	0.010 ± 0.000	0.016 ± 0.040

Cu, S, Fe, Mn (mg/100 g) content in SM, T1, T2, and T3 at initial, 2X, 3X Final concentration, and permeate; SM processed in UF at 15 °C, T1 = Homogenized WM processed in UF at 15 °C; T2 = Non-homogenized WM processed in UF at 15 °C; T3 = Non-homogenized WM processed at 43 °C.

## Data Availability

The original contributions presented in the study are included in the article, further inquiries can be directed to the corresponding author.
